# Supplementary material for: Causal Relationship between Aging and Anorexia Nervosa: A White-Matter-Microstructure-Mediated Mendelian Randomization Analysis
Source: Biomedicines. 2024 Aug 16;12(8):1874. doi: 10.3390/biomedicines12081874 (PMC11351342; doi:10.3390/biomedicines12081874)
Supplement: Supplementary file 1 [file biomedicines-12-01874-s001.zip › Supplementary Figure/Figure S8/Leaveoneout plot of MR analysis for TL to ubm-b-1573.pdf]

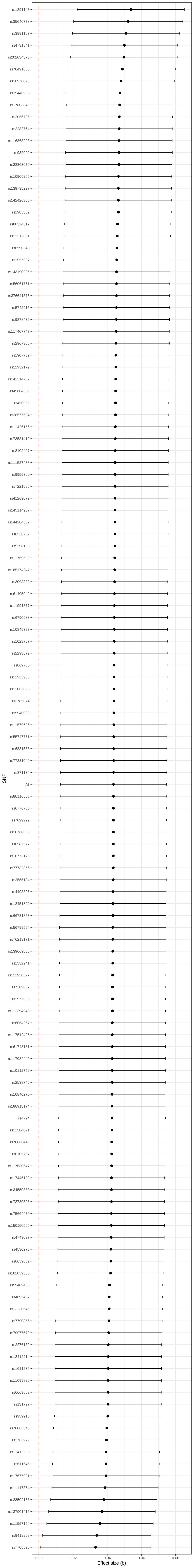
rs1291143
rs35640778
rs3891167
rs4731541
rs202034370
rs78491606
rs16978028
rs35446936
rs17803849
rs2056726
rs2282764
rs116863223
rs932002
rs28363070
rs10905255
rs139795227
rs142426306
rs1985369
rs80324517
rs11212631
rs6590343
rs1957917
rs143190905
rs56061761
rs376641875
rs5742915
rs9878436
rs117407747
rs2967355
rs1907702
rs12932179
rs141214782
rs45604339
rs450962
rs28577594
rs11426156
rs73581419
rs8102497
rs111527438
rs9955360
rs7221585
rs41269079
rs145114957
rs144204502
rs6536702
rs9398196
rs11769630
rs185174247
rs3093888
rs61405042
rs11991877
rs6790988
rs10845387
rs1023767
rs2293579
rs869785
rs12925933
rs13062095
rs3785074
rs9940099
rs11579626
rs55747751
rs6881568
rs77231040
rs871134
All
rs80116508
rs6776756
rs7099229
rs10768683
rs6587577
rs10773176
rs77732866
rs2555104
rs4498805
rs12451892
rs66731853
rs56799554
rs76219171
rs139669835
rs1332941
rs111950327
rs7209057
rs2977608
rs112394943
rs6054257
rs117512405
rs61748181
rs117034449
rs10112752
rs2538745
rs10840270
rs188918174
rs4724
rs11584821
rs76666449
rs8105767
rs117630647
rs17445108
rs34550383
rs73730598
rs75664430
rs150150565
rs4743037
rs4530278
rs6659669
rs182059586
rs59409453
rs4695407
rs13230646
rs7790856
rs79977579
rs2276182
rs12412214
rs1611236
rs11699829
rs6669563
rs131797
rs939916
rs76065543
rs2763979
rs11412296
rs611646
rs17677991
rs11117354
rs28502153
rs137901416
rs11557154
rs9419958
rs7705526
SNP
0.00
0.02
0.04
0.06
0.08
Effect size (b)
